# Omalizumab for cholinergic pruritus in a renal transplant and hemodialysis patient^؆^

**DOI:** 10.1016/j.abd.2025.501167

**Published:** 2025-08-04

**Authors:** Qing Shu, Xiang Zhong, Lixia Zhang, Qian Wang

**Affiliations:** a Institute of Dermatology and Venereology, Sichuan Provincial People's Hospital, School of Medicine, University of Electronic Science and Technology of China, Chengdu, China; b Department of Nephrology and Institute of Nephrology, Sichuan Provincial People’s Hospital, School of Medicine, University of Electronic Science and Technology of China, Sichuan Clinical Research Center for Kidney Diseases, Chengdu, China

*Dear Editor,*

Cholinergic Pruritus (CholP) is a rare condition characterized by generalized itching, tingling, or burning sensations without any skin lesions, triggered by increased body core temperature, and reversible after cooling. CholP is often resistant to antihistamines H1 (anti-H1), and the choice of medication is even more limited in patients with severe systemic disease. The efficacy and safety of omalizumab in anti-H1-refractory chronic spontaneous urticaria have been widely recognized, and it has also been successfully used in the treatment of Cholinergic Urticaria (CholU),^1^, ^2^ but there is little information regarding to this drug for the treatment of patients with concomitant immunosuppression and hemodialysis. Herein, we describe a case of omalizumab use as a treatment for CholP in a renal transplant and hemodialysis patient.

A 45-year-old male presented to our department, with complaints of generalized itching and tingling for more than 2-months. The unpleasant sensation triggered by increased body heat (e.g., hot baths, emotionally charged and taking spicy food) and relieved by cooling. It occurred several times a day and lasted for a few to 10 min each time but without any lesions. He has been taking oral tacrolimus and mycophenolate mofetil for 12-years after renal transplantation and hemodialysis 3 times a week was initiated more than 1-year ago. Since dialysis, he perspired less. Laboratory tests showed normal eosinophils, Immunoglobulin E (IgE), anti-Thyroperoxidase Antibody (anti-TPO Ab), and anti-Thyroglobulin Antibody (anti-Tg Ab). According to the patient's clinical manifestation, CholP, Chronic Kidney Disease-associated Pruritus (CKD-aP) and Acquired Idiopathic Generalized Anhidrosis (AIGA) were considered. However, patients with CKD-aP usually have persistent itching, which is severe at night as compared to daytime. Various factors such as showering, dialysis, heat, pressure, and cold can exacerbate itching, rather than just being induced by increased body core temperature.^3^ AIGA is characterized by anhidrosis/hypohidrosis without any apparent causes and is often troubled by tingling dermal pain upon sweating but not itching.^4^ Therefore, the final diagnosis is CholP. Considering the physical condition of the patients, we did not perform exercise challenge tests.

He was given cetirizine 10 mg qd, which provided partial relief in the first 2 weeks, but the effect gradually declined. Then, loratadine 10 mg daily was prescribed but was discontinued because of palpitation. Omalizumab (300 mg/4 weeks) in combination with cetirizine 10 mg qd was used with overall consideration of his underlying disease and drug accessibility and affordability. At the baseline, he had an itch Numeric Rating Scale (NRS) of 8/10, an Urticaria Control Test (UCT) score of 1/16, and a Dermatology Life Quality Index (DLQI) score of 12/30. Fortunately, the patient reported an obvious resolution after 4 weeks of treatment. He reached complete remission at 12 weeks (itch NRS of 0‒1/10, UCT score of 16/16, DLQI score of 2/30). However, at 24 weeks, there was some recurrence of symptoms, but still showed significant improvement from baseline (itch NRS of 4/10, UCT score of 12/16, DLQI score of 6/30). Table 1 shows the clinical scores at different time points. The patient did not report any adverse effects and there was no change in his immunosuppressive drugs and hemodialysis regimen.

**Table 1:** The clinical scores at baseline and 12‒24 weeks after omalizumab treatment.

Time points	Clinical scores		
	itch NRS	UCT	DLQI
Baseline	8	1	12
Week 12	0‒1	16	2
Week 24	4	12	6

NRS, Numeric Rating Scale; UCT, Urticaria Control Test; DLQI, Dermatology Life Quality Index.

CholP is a rare variant of Cholinergic Urticaria (CholU) without any lesions.^5^ The main treatment remains anti-H1, but it is usually less effective.^5^ Omalizumab, a recombinant humanized monoclonal antibody that inhibits IgE, has been approved for the treatment of chronic spontaneous urticaria. In recent years, there have been few reports of omalizumab for the treatment of CholP,^6^, ^7^ and reports in patients with combined renal transplantation and hemodialysis are lacking. Omalizumab has a relatively good safety profile, with rare serious adverse reaction such as anaphylactic shock. Since omalizumab is removed primarily by the reticuloendothelial system, it is less likely to affect renal function.

This CholP patient, with a history of renal transplantation and undergoing hemodialysis, had a good response and tolerance to omalizumab. Omalizumab did not have a notable impact on his renal function and relative medications. This case confirms the efficacy of omalizumab in CholP and seems to support that omalizumab treatment can be practiced in renal transplant and hemodialysis patients without apparent interference with the efficacy and safety of the drug. This may provide some reference for the use of omalizumab in patients with concomitant transplant status and/or hemodialysis. However, a longer follow-up period, as well as larger studies are needed to validate findings.

## Financial support

None declared.

## Authors’ contributions

Qing Shu: Data collection, analysis and interpretation of data; preparation and writing of the manuscript.

Xiang Zhong: Data collection, analysis and interpretation of data; preparation and writing of the manuscript.

Lixia Zhang: Critical review of the literature; approval of the final version of the manuscript.

Qian Wang: Study conception and planning; critical review of the literature; approval of the final version of the manuscript.

## Conflicts of interest

None declared.
